# Patient-derived scaffolds as a drug-testing platform for endocrine therapies in breast cancer

**DOI:** 10.1038/s41598-021-92724-9

**Published:** 2021-06-25

**Authors:** Anna Gustafsson, Elena Garre, Maria Carmen Leiva, Simona Salerno, Anders Ståhlberg, Göran Landberg

**Affiliations:** 1 Department of Laboratory Medicine, Institute of Biomedicine, Sahlgrenska Academy, Sahlgrenska Center for Cancer Research, University of Gothenburg, 41390 Gothenburg, Sweden; 2 Wallenberg Center for Molecular and Translational Medicine, University of Gothenburg, 41390 Gothenburg, Sweden; 3 Department of Clinical Genetics and Genomics, Sahlgrenska University Hospital, 41390 Gothenburg, Sweden

**Keywords:** Biological techniques, Cancer, Cell biology, Drug discovery, Molecular biology

## Abstract

Three-dimensional cell culture platforms based on decellularised patient-based microenvironments provide in vivo-like growth conditions allowing cancer cells to interact with intact structures and components of the surrounding tissue. A patient-derived scaffold (PDS) model was therefore evaluated as a testing platform for the endocrine therapies (Z)-4-Hydroxytamoxifen (4OHT) and fulvestrant as well as the CDK4/6-inhibitor palbociclib, monitoring the treatment responses in breast cancer cell lines MCF7 and T47D adapted to the patient-based microenvironments. MCF7 cells growing in PDSs showed increased resistance to 4OHT and fulvestrant treatment (100- and 20-fold) compared to 2D cultures. Quantitative PCR analyses of endocrine treated cancer cells in PDSs revealed upregulation of pluripotency markers further supported by increased self-renewal capacity in sphere formation assays. When comparing different 3D growth platforms including PDS, matrigel, gelatin sponges and 3D-printed hydrogels, 3D based cultures showed slightly varying responses to fulvestrant and palbociclib whereas PDS and matrigel cultures showed more similar gene expression profiles for 4OHT treatment compared to the other platforms. The results support that the PDS technique maximized to provide a multitude of smaller functional PDS replicates from each primary breast cancer, is an up-scalable patient-derived drug-testing platform available for gene expression profiling and downstream functional assays.

## Introduction

Breast cancer varies in terms of architecture and cellular composition^1^. Despite classifications of breast cancer into potentially clinically relevant subgroups, patients within each prognostic category can have distinctly different disease outcomes and therapeutic responses^2,3^. Approximately 70% of all breast cancer patients have estrogen receptor-α (ERα) positive tumors, and are therefore often suited for endocrine therapies^4^, such as the ERα-inhibitor tamoxifen and the selective ERα-degrader fulvestrant. Endocrine therapies target and inhibit ERα-dependent signaling thereby inducing proliferative arrest and apoptosis^5^. Novel targeting therapies for breast cancer are inhibitors of cyclin-dependent kinases (CDKs), which are key players of the cell cycle that is commonly dysregulated and activated in cancer. The CDK4/6 inhibitor palbociclib can further be used in combination with anti-estrogens and this parallel inhibition of the cell cycle and the ERα-signaling is an efficient treatment strategy for certain subgroups of breast cancer patients^6^. The exact molecular functions for palbociclib in vitro are nevertheless still under evaluation^7^. Despite general endocrine therapy benefits for breast cancer patients, resistance to the treatment occurs^8^. One possible explanation for the lack of effect for the anti-estrogens could be that the tumor-initiating subpopulation of cancer stem cells are mainly ERα-negative and will therefore not be targeted by conventional anti-estrogen therapies in ERα-positive cancer^9,10^. Several studies have also suggested that endocrine therapies can enrich for these cellular characteristics in breast cancer^11–13^.

Currently, the majority of pre-clinical cancer research is performed using two-dimensional (2D) cell culture systems that do not provide cell-cancer microenvironment connections, and therefore poorly recapitulate the complexity of cancers growing in vivo^14,15^. Three-dimensional (3D) cell culture models offer a noticeable advantage over the traditional 2D systems by providing a less artificial growth architecture, also inducing phenotypical features of the cancer cells. In vivo mice models such as patient-derived xenograft models have contributed substantially to translational research^16^. These models are nevertheless expensive, labor-intensive, and inefficient for the large-scale screenings required for personalized drug-discovery^17^. Novel 3D-systems such as organoid cultures display tumor heterogeneity in vitro and can be used in large-scale studies. Yet, organoids are dependent on added biochemical signals and can lack a functional microenvironment^18^. Consequently, there is a need for novel methods taken the influence of the cancer microenvironment on cellular responses into account when modelling cancer in vitro. To address this issue, several scaffold-based techniques that can be used for large-scale drug screening studies have emerged often using artificial biomaterials to create a surrogate scaffold for the cancer cells. Artificial scaffolds do not include tumor-specific characteristics^19^ whereas biological scaffold-based models using for example decellularised adipose or cancer tissues can provide cell-tissue interactions promoting in vivo-like features^20–23^. We have previously described how cancer cell lines can infiltrate and grow in decellularised patient-derived scaffolds (PDSs), generated from primary breast cancer samples^24^. Cellular growth and behaviors were highly influenced by the specific microenvironments influencing major properties as EMT and cancer stem cell features^25,26^. The adaptation of cancer cells growing in the PDS also mirrored clinical properties and the PDS-system therefore seems to be able to reproduce and monitor disease complexity in an in vitro setting, and could potentially also be suitable for drug testing^27–29^. Here, we have described the refinement of the PDS model to suit anti-cancer drug validations and further analyzed the effect of endocrine therapies and the CDK4/6-inhibitor palbociclib in PDS cultures using ERα-positive cancer cell lines. The PDS system was also compared to other commonly used 3D culture techniques and, unique properties of the PDS system was defined facilitating the development of clinically relevant approaches for anti-cancer drug development.

## Results

### Cryosectioned patient-derived scaffolds produce a high amount of functional replicates that support cellular growth and infiltration

**Figure 1:**
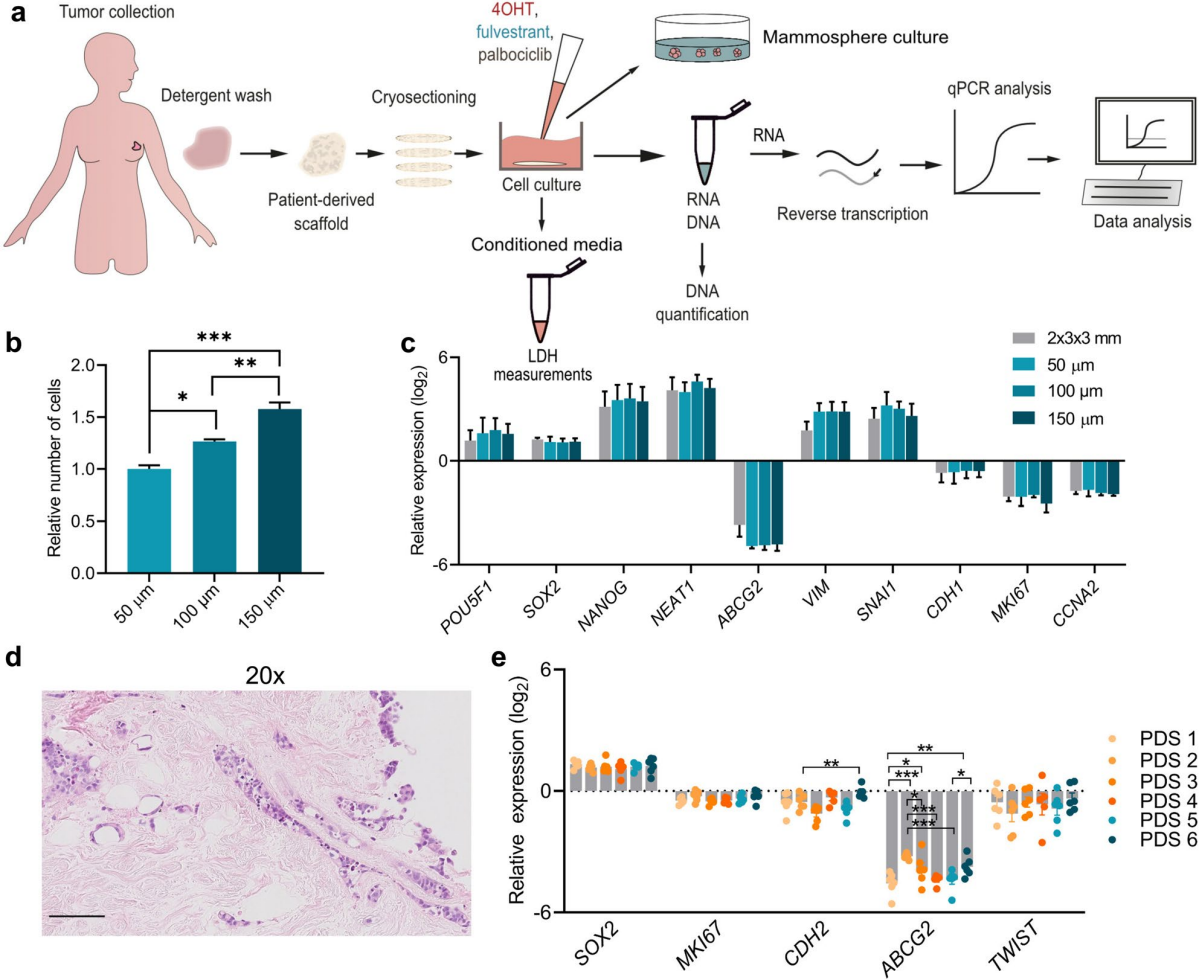
Size optimization of patient-derived scaffolds to enable maximum number of downstream experiments. (**a**) A schematic overview depicting the different steps from tumor collection, experimental procedures to data analysis*.* (**b**) Viability of MCF7 cells after three weeks of growth in patient-derived scaffolds (PDSs) with three different thickness (50, 100 and 150 µm) using alamar blue. Data is represented relative to alamar blue levels of 50 µm slices. Mean + SEM is shown, n = 3 PDSs. One-Way ANOVA with Tukey’s multiple comparison test was done between all the groups combinations (*p ≤ 0.05, **p ≤ 0.01, ***p ≤ 0.001). (**c**) Gene expression of MCF7 cells cultured in PDSs with different thicknesses relative to the expression in two-dimensional (2D) cultures and expressed in log2-scale. Mean + SEM is shown, n = 3 PDSs. (**d**) Image showing a representative hematoxylin and eosin staining of a 4 µm cross-section from a 150 µm PDS slice cultured with MCF7 cells after three weeks of growth. Bar represents 100 µm. (**e**) Bar graph demonstrating gene expression analysis of MCF7 cells after growth in 6 different PDSs compared to 2D cultures and expressed in log2-scale. Individual PDSs are indicated by colors and each dot represent a PDS slice. Mean + SEM is shown, n = 5–6. Two-Way ANOVA with Tukey’s multiple comparison test was done between all the groups combinations (*p ≤ 0.05, **p ≤ 0.01, ***p ≤ 0.001). Additional genes are included in Supplementary Fig. 1.

Primary breast cancer samples obtained from surgery were exposed to a series of detergent-washing steps generating cell-free patient-derived scaffolds (PDSs) that were re-cellularised with ERα-positive MCF7 cells and then cultured for 21 days (Fig. 1a). To optimize and maximize the number of functional PDS replicates obtained from each piece of cancer tissue, we included a cryosectioning step before the re-cellularization. The optimum size for the PDS slices regarding cell growth support was evaluated in 50, 100 or 150 µm cell-free PDS sections. As expected, thicker PDS slices supported the growth for a higher number of cells, suggesting that cells infiltrated the entire tissue section (Fig. 1b). Independently of the PDS slice thickness, all scaffold sections induced similar PDS-specific gene expression changes using a gene panel delineating different cellular phenotypes and cancer-related biological processes (Fig. 1c). Both thin sections and PDS cubes (2 × 3 × 3 mm) showed gene expression changes with upregulation of pluripotency (*POU5F1, SOX2, NANOG* and *NEAT1*) and EMT markers (*VIM* and *SNAI1*), but decrease of proliferation marker genes (*MKI67* and *CCNA2*), and the differentiation marker *CDH1* as well as the breast cancer stem cell (BCSC) gene *ABCG2*. Hematoxylin and eosin staining of cross-sections of 150 µm PDS slices supported infiltrative cell growth and adaptation to the surrounding microenvironment (Fig. 1d). For the subsequent experiments, 150 µm slices with a diameter of 6 mm were selected for being optimal to manage experimentally as well as producing relevant PDS-based information. Using this protocol, the largest breast cancer sample included in the study (height of ~ 11.4 mm) produced 76 technical replicates.

To evaluate the intra-variability between the replicates from the same PDS and the inter-variability between distinct PDSs, gene expression was assessed in MCF7 cells cultured in 6 PDSs from different patients using 5–6 slices from each scaffold (Fig. 1e, Supplementary Fig. 1a–d). The majority of the analyzed genes showed similar expression between slices from the same PDS and only *TWIST* displayed high intra-variability for all 6 PDSs. Differences in cell numbers between different PDSs and slices did not affect later downstream expression analysis, as demonstrated by the lack of correlation between RNA yield from each sample and the induced expression of the proliferation genes *MKI67* and *CCNA2* (Supplementary Fig. 1e–g). Regarding inter-PDS variability, several genes showed similar expression changes between PDS1-6, including the proliferation marker *MKI67* and the pluripotency marker *SOX2* (Fig. 1e, Supplementary Fig. 1) whereas other genes such as *CDH2* and *ABCG2* showed substantial differences between PDSs. Taken together, the data indicated that PDSs induced differences in the expression of certain gene markers, but with low intra-scaffold variability, making PDS slices suitable for up-scaled testing of different therapeutic responses and the evaluation of specific cancer microenvironments.

### Patient-derived scaffold cultures demonstrate increased resistance to (Z)-4-Hydroxytamoxifen and fulvestrant treatment compared to two-dimensional cultures

**Figure 2:**
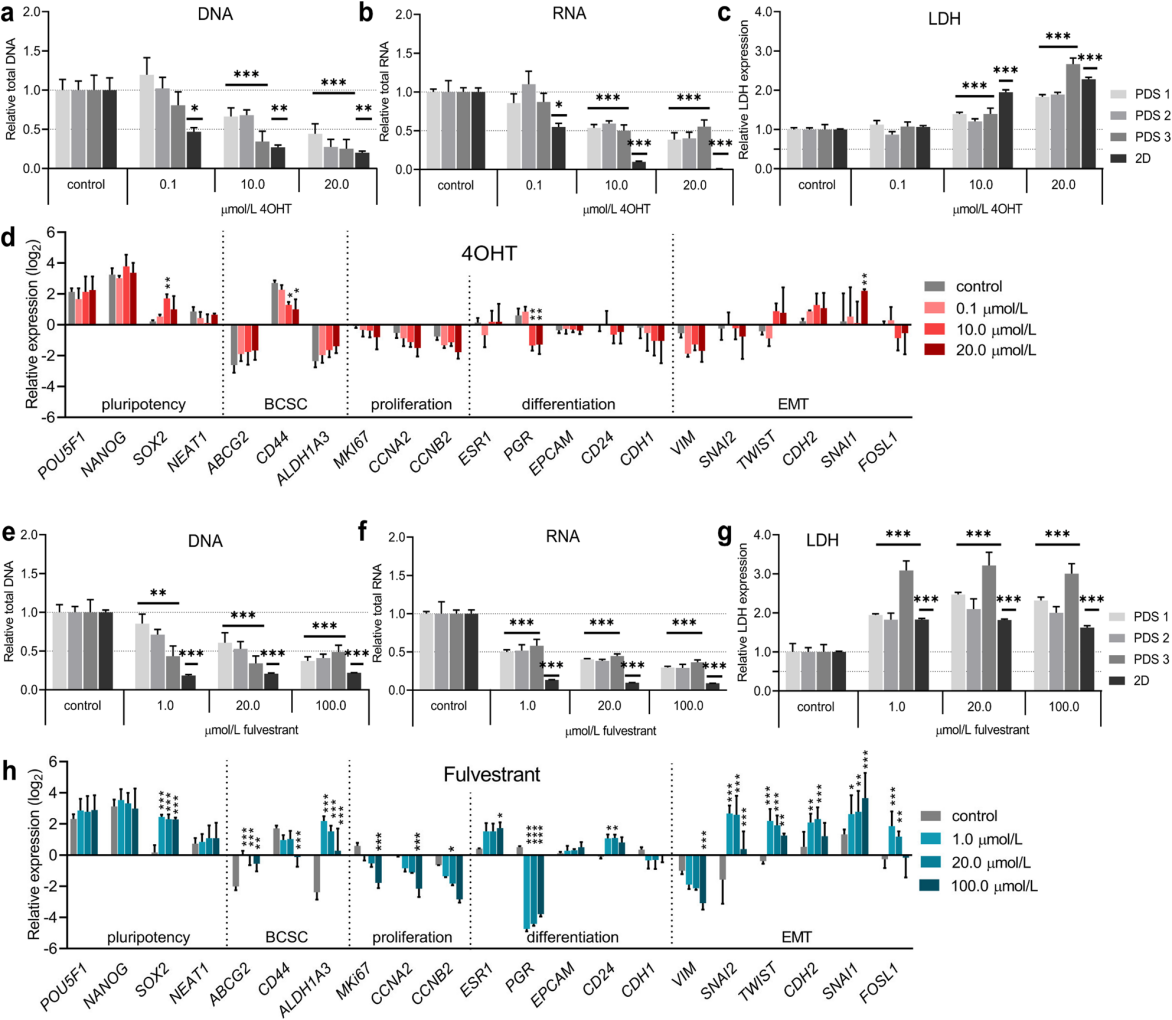
Cells cultured in patient-derived scaffolds show higher resistance to endocrine therapies than two-dimensional cultures. (**a**,**b**) Bar graphs showing relative amount of total DNA (**a**) and RNA (**b**) analyzed in cell lysates from patient-derived scaffolds (PDS) or two-dimensional (2D) cultures with MCF7 cells after 96 h treatment with either (Z)-4-Hydroxytamoxifen (4OHT) or vehicle controls. (**c**) Relative lactate dehydrogenase (LDH) levels in conditioned media after 96 h of 4OHT treatment in PDS and 2D cultures. (**d**) Gene expression analyzes of PDS cultures after treatment with 4OHT and controls (with vehicle). (**e**,**f**) Relative amount of total DNA (**e**) and RNA (**f**) analyzed in cell lysates from PDSs or 2D cultures with MCF7 cells after 96 h of treatment with either fulvestrant or vehicle controls. (**g**) Relative lactate dehydrogenase (LDH) levels in conditioned media after 96 h of fulvestrant treatment from PDS and 2D cultures. (**h**) Gene expression analyzes of PDS cultures after treatment with fulvestrant and controls (with vehicle). Mean + SEM (n = 3) is shown for all data. RNA, DNA and LDH levels are relative to their own control samples, and Student’s t-test was done between the treatment group and the control group for each data set (*p ≤ 0.05, **p ≤ 0.01, ***p ≤ 0.001). Gene expression data are relative to untreated 2D cultures, expressed in log2-scale, and Two-way ANOVA with Dunnett’s multiple comparisons test was done between the treatment group and the control group for each data set (*p ≤ 0.05, **p ≤ 0.01, ***p ≤ 0.001). BCSC; breast cancer stem cells, EMT; epithelial-to-mesenchymal transition.

Next, PDS cultures were tested as platform for endocrine therapies. MCF7 cells were cultured for 15 days using 150 µm PDS slices and complete DMEM culture media, followed by 24 h of serum starvation with 5% charcoal starved serum (CSS) and phenol-red free media, 24 h of 1% CSS supplemented DMEM and 96 h of treatments with the endocrine therapies tamoxifen’s derivate (Z)-4-Hydroxytamoxifen (4OHT) or fulvestrant in 1% CSS supplemented DMEM. In parallel, 2D cultures were seeded 24 h before starting the serum starvation steps and were treated in a similar way as the PDS cultures. After treatments, cell numbers in the PDS and 2D cultures were estimated by quantifying total DNA and RNA amount in the MCF7 cell lysates (Fig. 2a,b,e,f), whereas released lactate dehydrogenase (LDH) in conditioned media was used to assess cell death (Fig. 2c,g). Data indicated that 4OHT concentrations had to be increased 100-fold (from 0.1 to 10 µmol/L) in order to significantly reduce both DNA and RNA yields in the PDS cultures, compared to the IC50 calculated in 2D cultures (Fig. 2a,b, Supplementary Fig. 2a). The 4OHT toxicity in the PDS cultures was further dose-dependent, as illustrated by a gradual reduction of DNA and RNA levels and rise of released LDH with increasing drug concentrations (Fig. 2c) although with some variability between the different PDSs. Gene expression changes in MCF7 cells after 4OHT treatment in 2D cultures showed notable responses for several genes (Supplementary Fig. 3), whereas fewer gene expression changes were observed in PDS cultures (Fig. 2d) and only using the higher concentrations also inducing cytotoxic effects (10 and 20 µmol/L). High 4OHT concentration in MCF7 cells cultured in PDSs produced up to four-fold increase in *SOX2* and *SNAI1* expression and similarly reduced expression of *CD44* and *PGR* compared to PDS controls, suggesting that surviving cancer cells were enriched for EMT characteristics and were potentially more dedifferentiated.

In contrast, fulvestrant treatments significantly decreased DNA and RNA levels in the PDS cultures at the same concentration (1 µmol/L) that reduced growth to 50% in 2D cultures (Fig. 2e,f, Supplementary Fig. 2b), although the effect in 2D cultures was more pronounced. Up to 20 µmol/L fulvestrant was needed in order to reduce cell viability by 50% in the PDS cultures. Lactate dehydrogenase levels were elevated at all tested concentrations compared to controls (Fig. 2g), with large variations between individual PDSs, where PDS3 showed the largest decrease in DNA levels and also displayed the highest LDH release. Compared to 4OHT, fulvestrant treatment induced more pronounced gene expression changes in both PDS and 2D cultures and to some extent more similarities between the two cultures systems (Fig. 2h, Supplementary Fig. 4). For the PDS cultures, fulvestrant reduced the expression of *PGR* by 24–32 fold compared to PDS controls and also reduced the expression of several proliferation markers in a dose-dependent manner. In parallel, there was an increased expression of the pluripotency marker *SOX2* as well as several EMT-markers, suggesting an enrichment of cells with cancer stem cell characteristics after treatment.

The enrichment of cancer cells with pluripotency features after treatments with 4OHT and fulvestrant in PDS cultures as depicted by gene expression analysis, was further supported by an increase in Sox2 protein levels, and a reduction in PgR and Ccna2 proteins levels (Supplementary Fig. 5).

### The CDK4/6 inhibitor palbociclib decreased the expression of proliferation associated genes in patient-derived scaffold cultures

**Figure 3:**
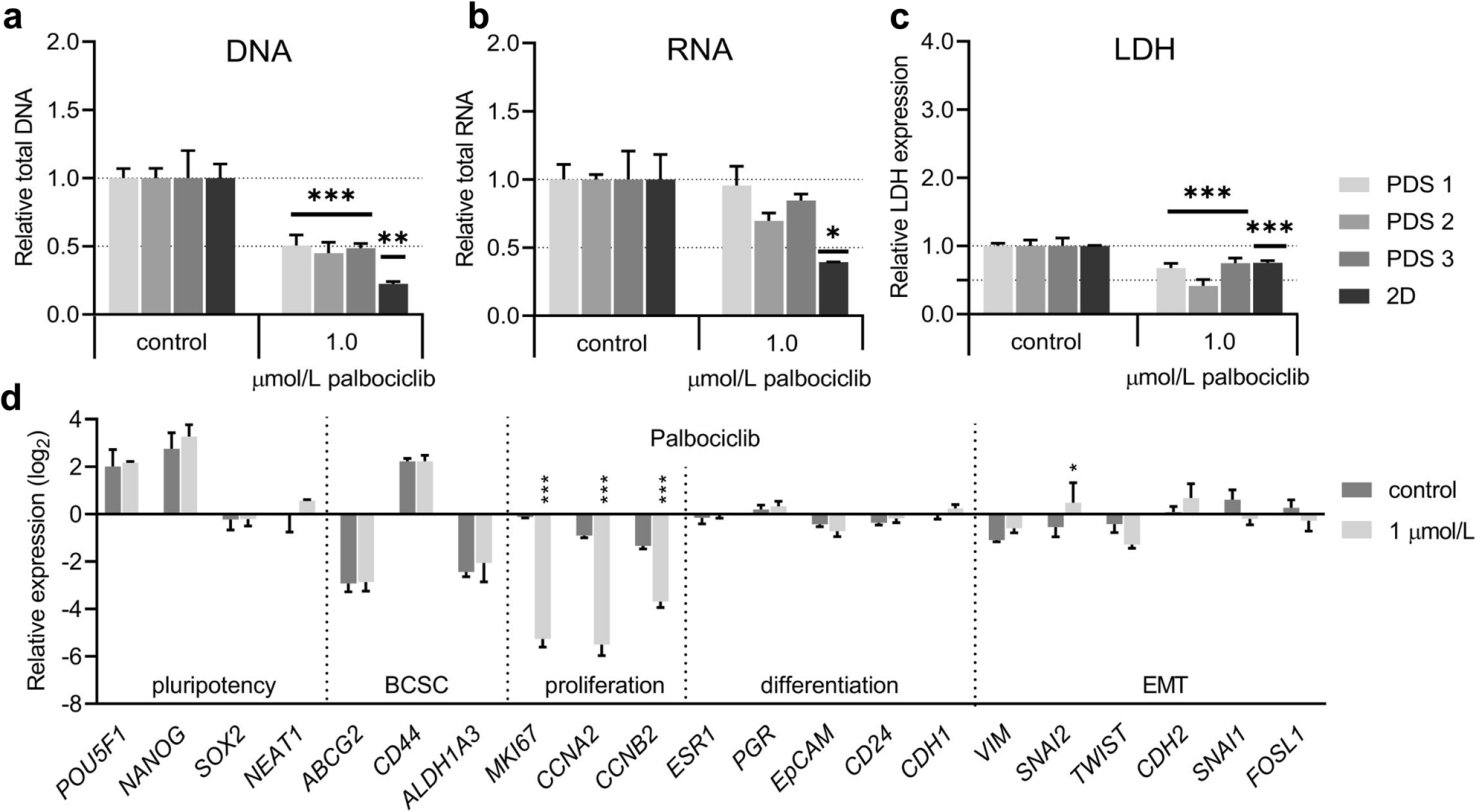
Treatment with palbociclib of cells cultured in patient-derived scaffolds and two-dimensional cultures. (**a**,**b**) Relative amount of total DNA (**a**) or RNA (**b**) analyzed in cell lysates generated from patient-derived scaffolds (PDS) or two-dimensional (2D) cultures with MCF7 cells after 96 h of treatment with either palbociclib or vehicle. (**c**) Relative lactate dehydrogenase (LDH) levels in conditioned media after 96 h of palbociclib treatment from PDS and 2D cultures. (**d**) Gene expression analyzes of PDS cultures after treatment with palbociclib and controls (with vehicle). Mean + SEM (n = 3) is shown for all data. RNA, DNA and LDH levels are relative to their own control samples, and Student’s t-test was done between the treatment group and the control group for each data set (*p ≤ 0.05, **p ≤ 0.01, ***p ≤ 0.001). Gene expression data are relative to untreated 2D cultures, expressed in log2-scale, and Two-way ANOVA with Dunnett’s multiple comparisons test was done between the treatment group and the control group for each data set (*p ≤ 0.05, **p ≤ 0.01, ***p ≤ 0.001). BCSC; breast cancer stem cells, EMT; epithelial-to-mesenchymal transition.

In addition, we investigated how PDS cultures were affected by the selective CDK4/6-inhibitor palbociclib that can be used in combination with endocrine therapies but not influencing ERα-signaling. After testing increasing concentrations of palbociclib in 2D cultures, a 50% reduction in growth and an increase up to 86% of G_0_/G_1_-phase cells was observed using 1 µmol/L of the drug (Supplementary Fig. 2c, Supplementary Fig. 6). Treatment of PDSs with 1 µmol/L palbociclib also reduced total DNA and LDH levels by 50% (Fig. 3a,c), whereas there was no significant changes in total RNA yields (Fig. 3b). As previously observed for the endocrine treatments, changes in gene expression after palbociclib treatments were again slightly more pronounced in 2D cultures compared to PDS cultures (Supplementary Fig. 7) but following similar trends. For the PDS cultures, the most notable effect was a strong reduction of the proliferation markers *MKI67, CCNA2, CCNB2* (16–64 fold) in palbociclib treated cells compared to controls (Fig. 3d, Supplementary Fig. 7), which was similar for 2D cultures as well. These results indicate that palbociclib inhibited proliferation in PDS cultures to a similar extent as in 2D cultures.

### Treatment with (Z)-4-Hydroxytamoxifen and fulvestrant in patient-derived scaffold cultures caused an enrichment in cells with cancer stem cell properties

**Figure 4:**
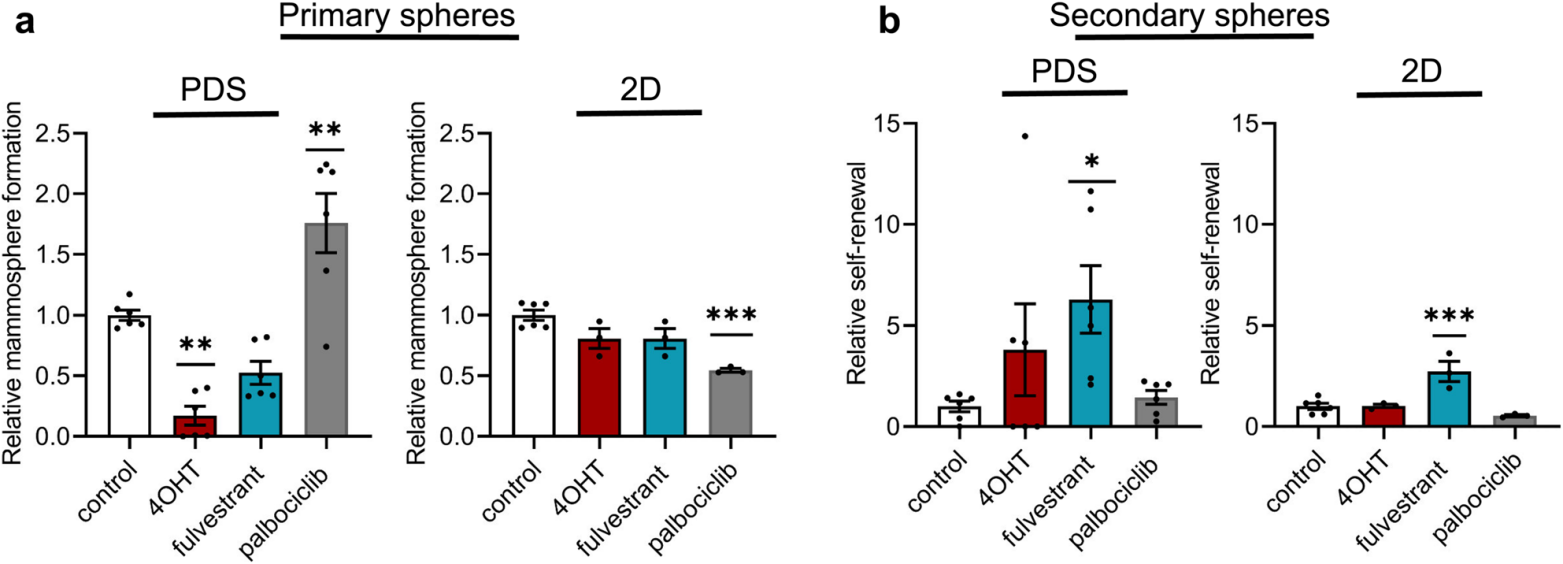
Endocrine treatments in patient-derived scaffolds decrease sphere formation capacity but enrich for cells with a higher capacity to self-renew. (**a**) Mammosphere formation assay quantifying MCF7 cells ability to form spheres after treatment with either 10 µmol/L or 0.1 µmol/L (Z)-4-Hydroxytamoxifen (4OHT) for patient-derived scaffold (PDS) and two-dimensional (2D) cultures, respectively, 20 µmol/L or 1 µmol/L fulvestrant for PDS and 2D cultures, respectively or 1 µmol/L palbociclib, compared to controls. (**b**) Ability of cells from primary spheres to self-renew and generate secondary spheres compared to controls. Mean + SEM is shown, (N_PDS_ = 6, where each dot represents the average of 3 slices from each PDS grown with different cell passages, and N_2D_ = 3). One-way ANOVA with Dunnett’s multiple comparisons test was done between the treatment group and the control group (*p ≤ 0.05, **p ≤ 0.01 ***p ≤ 0.001).

To further evaluate the influence of the PDS microenvironment on cancer stem cell properties after drug treatments, we utilized the mammosphere assay as a surrogate measurement for cancer stem cell activity of cells grown and treated in PDSs and compared to 2D cultures. Cells grown in PDSs for a total of 21 days, including serum starvation steps and drug treatments, were dissociated from the PDSs or 2D cultures, prepared into single-cell suspensions and cultured in non-adherent conditions. 4OHT treatment of cells growing in PDSs significantly blocked the mammosphere forming capacity (Fig. 4a), while fulvestrant treatment caused a slight reduction in mammospheres. In contrast, there were no changes in sphere-forming capacities using endocrine treated cells from 2D cultures. In order to determine the potential long-term effects of the drugs, we dissociated the primary spheres and generated secondary spheres (Fig. 4b). The results indicated that cells from PDSs treated with 4OHT and fulvestrant increased their secondary sphere formation capacity by 4- and 6-fold. For the 2D cultures, only fulvestrant treatment increased the secondary sphere formation capacity (3-fold). Interestingly, the proliferation-inhibitor palbociclib significantly increased the number of primary mammospheres from PDS cultures by more than 50%, while the mammosphere formation capacity decreased in 2D cultures (Fig. 4a). Palbociclib treatment, however, did not influence long term self-renewal capacity of the cells from either cell culture condition (Fig. 4b).

### Cancer cells response is heavily influenced by drug treatments as well as cell culture techniques

**Figure 5:**
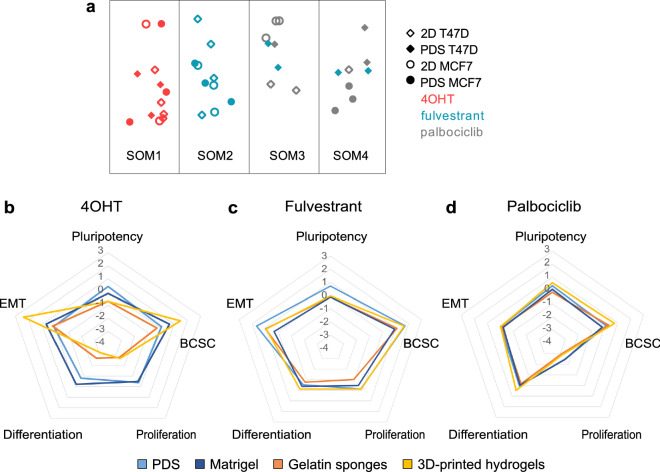
Evaluation of induced gene expression in different three-dimensional growth platforms after drug treatments. (**a**) Self-organizing Map (SOM) analysis of the gene expression data for T47D cells (diamonds) or MCF7 cells (circles) treated with 4OHT (red), fulvestrant (blue) or palbociclib (grey) in 2D cultures (open symbols) or PDSs (filled symbols). Gene expression values used for SOM are expressed in log2-scale and relative to their own untreated controls (vehicle), 3–4 biological replicates of each condition and cell line are included. (**b**–**d**) Radar charts show the main trend of the gene expression changes in pluripotency, breast cancer stem cell (BCSC), proliferation, differentiation and epithelial-to-mesenchymal transition (EMT) categories for MCF7 cells grown in patient-derived scaffolds (PDS), gelatin sponges, matrigel and three-dimensional (3D)-printed hydrogels, after treatment with (**b**) 10 µmol/L (Z)-4-Hydroxytamoxifen (4OHT), (**c**) 20 µmol/L fulvestrant or (**d**) 1 µmol/L palbociclib. Gene expression for each culture platform is expressed relative to their own untreated control (vehicle). All plotted data in the radar graphs was calculated as the average of log2-scale expression of the genes grouped in each category (categories are detailed in Supplementary Table 1) and including 3 replicates for each culture platform.

To further delineate the response to the specific treatments using the PDS platform and 2D cultures, we next analysed gene expression changes in the ERα-positive cell line T47D using the same treatment conditions for 4OHT, fulvestrant and palbociclib as previously described for MCF7 cells. Self-organizing map (SOM) analysis of gene expression data from both cells lines showed that SOM groups were mainly formed based on treatments instead of cell lines and that the culture platform used (PDS or 2D) separated into different SOM groups for cells growing in PDSs treated with fulvestrant and palbociclib. These data indicate that treatment induced cell responses were to some extent similar for the two cell lines (Fig. 5a, Supplementary Fig. 8).

We next evaluated similarities of the treatment effects in PDS cultures with other available 3D growth models and investigated if PDS specific effects could be separated from those generally associated with 3D growth. MCF7 cells were therefore grown for 21 days in (1) PDS (2) 3D-printed hydrogels consisting of 10% alginate and 5% hydroxyapatite, (3) gelatin sponges or (4) matrigel cultures and expression changes compared to conventional 2D cultures were analyzed. In untreated cultures, radar chart plotting the trend of gene expression changes in pluripotency, breast cancer stem cell (BCSC), proliferation, differentiation and epithelial-to-mesenchymal transition (EMT) categories (detailed in Supplemental Table 1) clearly illustrated that the gene expression profiles of PDSs were distinguished from the other models, and all 3D models were distinctly different from 2D cultures (Supplementary Figs. 9 and 10a).

Drug treatments with 4OHT, fulvestrant or palbociclib of the different 3D cell culture systems were also performed. As illustrated by the radar charts, 4OHT treated PDSs and matrigel cultures induced similar mild changes in gene expression, while gelatin sponges and 3D-printed hydrogels instead showed pronounced and general downregulation of proliferation and differentiation genes (Fig. 5b, Supplementary Fig. 10b). Interestingly, both fulvestrant (Fig. 5c, Supplementary Fig. 10c) and palbociclib (Fig. 5d, Supplementary Fig. 10d) treatments induced similar gene expression changes in all the 3D models. However, fulvestrant treated PDSs also influenced the gene expression of additional pluripotency and EMT genes compared to the other models (Supplementary Fig. 10c).

## Discussion

There is an obvious need for more relevant model systems in cancer research and drug development. Yet, proper 3D systems taking the cancer microenvironment into consideration are still lacking^27^. We therefore evaluated and streamlined the drug screening potential of a recently described PDS model that preserves the cell-free cancer microenvironment including structure and proteins^24^. We optimized the sectioning of PDSs into thinner slices that showed appropriate experimental stability and robustness, allowing an increase of the replicate numbers obtained from each individual PDS, which is beneficial given that the access to patient material is often limited. Importantly, replicates from the same PDS showed low intratumor heterogeneity, which is essential and allows evaluation of several drug treatments using the same specific patient samples. When studying the treatment responses we observed differences in drug sensitivity, gene expression profiles and induced spheres self-renewal capacities between PDSs from different breast cancer patients. These data support that unique microenvironments indeed influence cancer cells regarding important cancer properties such as therapy resistance and cellular differentiation. Besides supporting the physiological relevance of the PDS model for in vitro drug screening, the observed inter-PDS variability is in line with previously published data and is most likely the result of innate differences of the cancers such as cancer subtype, origin and cellular differentiation^24,30^. Future experiments using larger tumor cohort’s nevertheless need to be performed in order to better define the exact relation between the variations in the PDS adaptation as well the PDS drug treatment responses in relation to clinical responses. Co-cultures with other cell types, such as cancer-associated fibroblasts could also add immunological complexity to the system, while still maintaining the simplicity of the model to be able to use standardized analyzes such as qPCR.

Previously, it has been reported that 3D growth decreases the drug sensitivity for endocrine therapies in vitro compared to 2D cultures, as exemplified by increased resistance to tamoxifen and fulvestrant in ER + /HER2 + cell lines cultured in matrigel^31^, to fulvestrant in MCF7 cells growing in collagen scaffolds, and to tamoxifen in chitosan polymer scaffolds^32,33^. In line with these studies, higher concentrations of both 4OHT and fulvestrant were required to demonstrate cytotoxic properties in MCF7 cells grown in PDSs compared to 2D cultures. This increased drug resistance was also illustrated by the gene expression profiles, where 4OHT and fulvestrant treated PDSs showed less pronounced changes compared 2D cultures. Interestingly, the same concentration of palbociclib inhibited growth in both 2D and PDS cultures, suggesting that drug sensitivity in PDSs may also be dependent on the mechanistic actions of the compounds. Moreover, when introducing another ERα-positive cell line, T47D, to the PDS system it was clear that the drug effect was to a large extent the driver of the cellular response, more than the differences between cancer cell lines, which confirms the robustness of the system^14,15^. It is well known that cellular 3D growth contributes to more in vivo-like phenotypes^34^. However, the presented data indicated that different 3D models could induce different gene expression profiles of cancer cells in response to the treatments. Although there was only moderate differences between the various 3D models for fulvestrant and palbociclib treatments, 4OHT clearly induced different cellular phenotypes depending on the growth platform used. The fact that PDSs and matrigel cultures induced more similar gene expression profiles supports the importance of using cancer tissue-derived materials in drug testing. Since the expression of pluripotency markers were clearly affected differently by the various model systems, the link between the cancer microenvironment and the problematic population of cancer stem cells with pluripotency features indeed needs to be further clarified^34^.

Endocrine treatments in PDS cultures induced changes in gene expression mirroring cancer stem cell features. Interestingly, the pluripotency marker *SOX2* which has also been linked to increased resistance to endocrine therapies in vitro and in vivo^11,12,35^, was highly expressed in MCF7 cells growing in PDSs and exposed to 4OHT or fulvestrant treatment. Enrichment of cancer stem cell characteristics after endocrine treatment in PDSs was corroborated by the mammosphere assay, where MCF7 cells showed an increased capacity for self-renewal. The results indicated that 4OHT and fulvestrant treatments in PDSs initially reduced the number of viable cancer cells with ability to form spheres, but the remaining cells had high capacity to form secondary spheres. This may be due to that the mammosphere assay is also an indirect measurement of proliferation and cells from PDSs with cancer stem cell characteristics were low proliferative and may require prolonged time in order to be able to grow under non-adherent conditions. The observation that some of the PDSs induced higher self-renewal of spheres compared to other scaffolds after endocrine treatment further supports that some patient tumor microenvironments have a substantial capacity to promote cancer stem cell properties.

Given that palbociclib inhibits the CDK4/6 pathway, we expected this treatment to exclusively inhibit the expression of proliferation-related genes, but besides the general decrease in proliferation we observed that treatment in 2D cultures altered the expression of several other genes, suggesting that this drug induced less specific effects in 2D conditions compared to PDS cultures. In the sphere-forming assay, PDS-derived cells treated with palbociclib initially formed a higher number of primary spheres but this effect was not retained in secondary sphere forming assays. These data indicate that CDK4/6-blockage can influence cancer stem cell features, alternatively the anoikis process, under certain conditions. However, it is unlikely that the long-term effect of palbociclib treatment in PDSs would increase cancer stem cell properties or cells regenerative capacity. The initial increase in sphere formation after palbociclib treatment in PDSs may instead be influenced by strong proliferative inhibition by the drug followed by a temporary proliferative rebound effect that could be due to phosphorylation of the CDK4/6 target retinoblastoma protein by other CDKs, such as CDK2, that could cause cancer cells to enter in S-phase, despite inhibition by palbociclib^36^. In support of this, clinical trials have shown that Ki67 levels increased in some cancer patient biopsies in neoadjuvant window studies after drug washout before surgery, suggesting that the effect of palbociclib is reversible also in vivo^37,38^. This also highlights that sphere-forming assays should be complemented with other assays in order to correctly evaluate cancer stem cell activities.

The use of the PDS-platform in drug testing has potentially several advantages compared to other 3D models, since the scaffolds are derived from human tissues and include an imprint of the information from the microenvironment. Moreover, they are easy to handle since they do not require passaging, or additional growth substrates besides conventional cell culture media. The data presented indicate that 3D growth models and especially tissue-derived products, clearly influenced the treatment effects using different read-outs compared to simple models such as 2D growth systems or synthetic 3D growth platforms. Our data further support that PDSs infiltrated with cancer cell lines provide insight into how the cancer microenvironment influences the cell response to therapies and indeed support the usefulness and value of this model system as a pre-clinical tool prior to in vivo studies. Complex growth model systems will be crucial in order to monitor and model the inevitable influence of the cancer microenvironments on cancer treatment effects and therefore be fundamental for the discovery of novel anti-cancer therapies.

## Material and methods

### Patient material

Primary breast tumors from patients without neoadjuvant therapy were collected directly after surgery at the clinical pathology diagnostic unit at Sahlgrenska University Hospital (Gothenburg, Sweden). Material in this study and histo-pathological characteristics for samples are detailed in Supplementary Table 2. Collection and processing of patient data was approved by the Regional Ethics Committee (Regionala Etikprövningsnämnden) in Gothenburg (DNR: 515-12 and T972-18). All methods were conducted according to relevant ethical guidelines and regulations. The participants included in the study were in the age range of 37–84 years-old, and informed consents were obtained from all of them.

### Patient-derived scaffold preparations

Tumors were decellularised according to the previously described protocol^24^. In short, tumors were decellularised during 6 h with lysis buffer containing EDTA (0.5 mM; VWR), SDS (3.5 mM; AppliChem), PMSF solution (0.4 mM; Merck) and sodium azide (3.07 mM; G-Biosciences) followed by a rinse step with PBS (Medicago) supplemented with sodium azide, EDTA and PMSF solution for 15 min, repeated twice. PDSs were thereafter washed during 72 h with sterile H_2_O renewed twice a day, followed by 24 h of PBS to remove cellular debris. All steps were performed in 37 °C, 175 rpm (Incu-Shaker™ 10L, Benchmark). Then, PDSs were placed in a storage solution, containing sodium azide, EDTA and dH_2_O at 4 °C, for preservation until use. For cryosectioning, PDSs were cut into even sizes using biopsy punch needles (Kruuse) to a diameter of 6 mm, snap-frozen in liquid nitrogen and sectioned to 50, 100 or 150 µm thick sections using CM3050S cryotome (Leica). The PDS slices were sterilized in PBS supplemented with 0.1% peracetic acid (EMD Millipore) for 1 h at room temperature, followed by PBS containing 1% Antibiotic–Antimycotic (Gibco) wash for 24 h, 37 °C at 175 rpm.

### Cell culture

MCF7 was cultured in DMEM (Gibco), supplemented with 10% FBS, 100 U/ml penicillin/streptomycin, 1% L-glutamine, 1% non-essential amino acids (all ThermoFisher Scientific) and 1% antibiotic-antimitotic (Gibco). T47D was cultured in RPMI; Gibco) with 10% FBS, 100 U/ml penicillin/streptomycin, 1% L-glutamine, 1% sodium-pyruvate (all ThermoFisher Scientific) and 1% antibiotic-antimitotic (Gibco). Cells were cultured in humidified chambers 37 °C, 20% O_2_, 5% CO_2_. All cells were bought and authenticated by ATCC, and confirmed to be mycoplasma negative.

### Patient-derived scaffold re-cellularisation

PDS slices were placed in 48-well plates and 3 × 10^5^ MCF7 cells or T47D cells were added to a final volume of 0.7 ml cell line-specific media. After 24 h, PDSs were transferred to new wells containing fresh media that was renewed weekly, for 21 days in total.

### Three-dimensional models

3D-printed hydrogels with alginate 10% (w/v; Protanal LF10/60) containing 5% hydroxyapatite (Sigma-Aldrich) were spread using digital T25 ULTRA-TURRAX Disperser (IKA) at 8000 rpm for 60 s, and rested in 4 °C until usage. Alginate and hydroxyapatite were then printed with a Bioplotter (Enviosiontech) (⌀15 mm × 2 mm; grid distance 1.5 mm, 90°) in 4 layers by using 400 µm extrusion needles, and 0.1 M CaCl_2_-spray was added to crosslink each layer. 3D-printed hydrogels were placed in complete DMEM before seeding with 3 × 10^5^ MCF7 cells to remove CaCl_2_. Porcine Spongostan Dental gelatin sponges (Ethicon) were directly seeded with 3 × 10^5^ MCF7 cells. Matrigel cultures were made by mixing equal parts growth factor reduced matrigel (Corning) together with DMEM on ice 30 min. The solution was poured into 24-well plates, and placed in 37 °C 30 min to set. Excess media was replaced with 3 × 10^5^ MCF7 cells. 3D-printed hydrogels and gelatin sponges were moved to new wells with fresh media after 24 h, while the media was exchanged on the matrigel cultures. This procedure was repeated 1–2 times per week to ensure fresh media, for a total of 15 days before initiation of serum starvation (total 48 h) and drug testing (96 h), as described below.

### Viability assay

Number of cells in PDSs with different thickness were determined using alamar blue. After 3 weeks of growth, MCF7-PDSs were incubated with 10% alamar blue solution (Invitrogen) for 2 h, afterwards PDS slices were removed and fluorescence was measured (BMG Omega Flostar). Media from 50 µm PDS slices were used as reference points. For concentration tests of drugs in 2D cultures, 5000 MCF7 cells were seeded in 96-well plates containing 5% CSS in phenol red-free DMEM/F12 (Gibco) and incubated for 24 h. Then, cells were stimulated with E2 (1 nmol/L; Sigma) and increasing concentrations of 4OHT, fulvestrant, or palbociclib (all Merck) in 1% charcoal starved serum (CSS) added for 96 h. Fluorescence was measured after 2 h of incubation with 10% alamar blue solution. Vehicle controls were used as reference points.

### Drug testing of two-dimensional and three-dimensional models

Before drug testing MCF7 and T47D 2D cultures were serum starved in phenol red-free DMEM/F12 (Gibco) or in phenol red-free RPMI (Gibco), respectively, supplemented with 5% charcoal starved serum (CSS) for 24 h, followed by 24 h in 1% CSS in DMEM or RPMI. CSS was prepared as previously described^39^. MCF7-PDSs and other 3D models were grown for 15 days, and T47D-PDSs were grown for 11 days before the serum starvation steps. To stimulate ERα-signaling, 17-β-estrodiol (1 nmol/L; Sigma) dissolved in EtOH (Solveco) was added to the cells together with 4OHT treatment dissolved in EtOH, fulvestrant dissolved in DMSO (Sigma) or palbociclib dissolved in sterile H_2_O (all drugs from Merck). Drug treatments were performed with 1% CSS + DMEM 96 h for MCF7 cells. T47D cells were treated for additional 96 h in fresh 1% CSS + RPMI at the same concentrations. All PDSs and 3D models were grown for a total of 21 days.

### RNA extraction, cDNA synthesis and qPCR

PDSs and 2D cultures were lysed using RLT buffer. Cells from matrigel cultures, gelatin sponges and 3D-printed hydrogels were lysed in Qiazol. All samples were stored in -80 °C until use. Thawed samples were homogenized with steal beads in TissueLyser II for 5 min × 2 at 25 Hz, followed by centrifugation at 12,000 rcf for 5 min (all Qiagen). Qiazol-lysates were mixed with chloroform (Merck), followed by centrifugation at 12,000 rcf for 15 min at 4 °C. RNA was thereafter extracted from the supernatants using RNeasy Micro kit in a QIAcube device including DNAse digestion step, or AllPrep DNA/RNA/Protein Mini Kit. For DNA quantification using the Allprep kit, separate DNA columns were removed before DNAse digestion (all Qiagen). Both RNA and DNA concentrations were measured using NanoDrop (ThermoFisher Scientific). Quality of RNA was randomly evaluated by DNF-472 HS RNA (15 nt) using 5400 Fragment analyzer System (both Aglient) according to manufacturer’s instructions. 100–500 ng RNA supplemented with RNA Spike II (TATAA Biocenter) were transcribed using GrandScript cDNA synthesis kit (TATAA Biocenter) in T100 Thermal Cycler (BioRad) and diluted 1:5 in RNase/DNase-free H_2_O (ThermoFisher Scientific). Next, diluted cDNA was added to SYBR GrandMaster Mix (TATAA Biocenter), and reactions were performed in CFX384 Touch Real-time PCR Detection System (BioRad) using 400 nM of each primer (Sigma-Aldrich; detailed in Supplementary Table 1). The experimental temperature in the machine was 95 °C for 2 min, followed by 45 cycles of amplification at 95 °C for 5 s, 60 °C for 20 s, and 70 °C for 20 s and a melting curve analysis at 65–95 °C with 0.5 °C, per 5 s increase. All assays were evaluated by melting curve analysis and cycles of quantification were confined by regression with CFX Manager Software 3.1 (BioRad). Data pre-processing were performed in GenEx ver 7 (MultiID, https://www.multid.se). Non-template controls were included and amplification of gDNA was ruled out using reverse-transcriptase-free controls in the production of cDNA. Cycles of quantification values larger than 35 were substituted with 36, and missing values were replaced with the imputation of PDS slice replicates. Reference genes were identified with NormFinder algorithm. Gene expression was expressed in relative quantities to 2D or PDS controls and log2 transformed. These experiments were performed according to the Minimum Information for Publication of Quantitative Real-Time PCR Experiments (*MIQE*) guidelines^40^.

### Mammosphere culture

Mammosphere assay was performed after 21 days of growth, including 2 days of serum starvations and 4 days of drug treatments at the concentrations which inhibited growth by 50% for 2D and 3D cultures respectively. After treatment, cells were washed with PBS and detached from plates or PDS slices using Accutase (Corning), at 37 °C for 10 min with shaking. Next, single-cell suspensions were made using a 25G needle. Cell numbers were assessed using Trypan blue (ThermoFisher Scientific) in Bürker-Türk chambers (VWR). Single-cell suspensions were seeded at 500 cells/cm^2^ in non-adherent polyHEMA (Sigma-Aldrich) coated culture plates in phenol red-free DMEM (Gibco) containing 1% penicillin/streptomycin, 2% B27 supplement (Gibco) and 20 ng/µl EGF (BD Bioscience). Following 5 days of incubation, mammospheres ≥ 50 μM were counted. Spheres were then dissociated and made into single-cell suspensions, seeded and secondary spheres were counted after 5 additional days. For PDS cultures, 6 different PDSs were included in the study with 3 slices each with different passages of MCF7 cells for each treatment. Two-dimensional culture replicates were made from 3 different passages of MCF7 cells for each treatment.

### Statistical analysis

Data was processed using GraphPad Prism 9.1.0 (https://www.graphpad.com/) and Excel 2016, and final figures generated in Affinity Designer 1.7.3.481 (https://affinity.serif.com/en-us/designer/). Student’s t-test and ANOVA were performed using GraphPad Prism 9.1.0. Self-organizing map (SOM) analyzes were applied to log2-scale gene expression data from drug treated samples relative to their own untreated control (vehicle) and performed in GenEx ver 7 (MultiID, https://www.multid.se). α = 0.05 and *p-*values < 0.05 were considered significant.

## Supplementary Information

Supplementary Information.

## Data Availability

The datasets generated and/or analyzed during the current study are available from the corresponding author on reasonable request.
