# Supplementary material for: Patient-derived scaffolds as a drug-testing platform for endocrine therapies in breast cancer
Source: Sci Rep. 2021 Jun 25;11:13334. doi: 10.1038/s41598-021-92724-9 (PMC8233392; doi:10.1038/s41598-021-92724-9)
Supplement: Supplementary file 1 — Supplementary Information. [file 41598_2021_92724_MOESM1_ESM.pdf]

Supplementary information

Supplementary Methods

Histology

Patient-derived scaffold sections with cells were fixated in 4% phosphate buffered formalin (Histolab) and dehydrated with increasing levels of EtOH (Solveco). Tissues were thereafter infiltrated with xylene and embedded in paraffin (all HistoLab) and sectioned into 4 µm slices using Microm Cool-cut (ThermoFisher Scientific). Next, the sections were deparaffinised and stained with Mayer's hematoxylin and 0.2% eosin. Subsequently, samples were dehydrated with increasing levels of EtOH and mounted onto glass slides with Pertex (all HistoLab).

Lactate dehydrogenase assay

Assessment of cell death was performed using Cytotoxicity Detection Kit (Roche) according to manufacturer's instructions by quantifying lactate dehydrogenase (LDH) from conditioned media. After treatment of PDS or 2D cultures, 100 µl conditioned media was mixed with 100 µl LDH reaction mix and fluorescence was measured after 30 minutes of incubation in Multi-mode reader (Biotek). Media from PDS control samples were used as reference points for the measurements.

Flow cytometry of cell cycle

Cell cycle distribution of cells were evaluated after 48 hours of treatment with palbociclib (1 nmol/L-2 µmol/L). Cells were harvested, washed in ice cold PBS (Gibco) and fixated with 70 % ethanol (Solveco). Subsequently, cells were incubated 30 min on ice. After centrifugation, pellet was re-suspended in 50 µl Ribonuclease A (100 µg/ml; Sigma-Aldrich) diluted in Mg_2/Cl_2-free PBS (Gibco) and thereafter, 200 µl propidium iodide were added (50 µg/ml; Sigma-Aldrich). Cells were incubated for 5-10 min at room temperature and DNA content was analyzed using flow cytometry (Accuri) at 488 nm. For each sample, 1 x 10^4 events were collected and the data was analyzed using FlowJo (TreeStar, Inc).

Wester Blotting

Protein samples were diluted in 5x Laemmli sample buffer (10 % SDS, 0.5 M Tris-HCl pH6.8, 0.5 % bromophenol blue, 10 % 2-β-mercaptoethanol) to a final concentration of 1x, and boiled for 10 min at 95 °C. Protein separation was performed through 4 – 20 % Mini-PROTEAN® TGX™ Precast Gel electrophoresis according to standard methods and using Bio-Rad Mini-

Protean II equipment (Bio-Rad). Protein was transferred to nitrocellulose membranes using the same equipment. Then, membranes were stained with SYPRO Ruby (Bio-Rad) to assess the efficiency of protein transfer to the blot and the loading. Membranes were blocked in 5 % (w/v) milk dissolved in 1x PBST buffer (137 mM NaCl, 2.7 mM KCl, 10 mM Na_2HPO_4, 1.8 mM KH_2PO_4; all from Sigma) for 1 hour at room temperature with shaking. Afterwards, primary antibodies appropriately diluted in blocking buffer were incubated with the membrane overnight at 4 °C with shaking. The membranes were rinsed three times with 1x PBST buffer, and then, incubated for 1 hour in the appropriate horseradish peroxidase conjugated secondary antibody diluted (1:5000) in blocking buffer. Membranes were then washed three times in 1x PBST with shaking and they were developed with SuperSignal™ West Femto Maximum Sensitivity Substrate (ThermoFisher). Films were scanned at 600 dpi and saved as TIFF files. Primary antibodies: anti-Sox2 (mouse, monoclonal, Abnova, 1:500), anti-Ccna2 (Recombinant Anti-Cyclin A2 antibody, EPR17351, rabbit, Abcam, 1:500), anti-PgR (mouse, clone PgR 636, 1:200) and anti-Snai1 (C15d3, rabbit, Cell Signaling, 1:500). Secondary antibodies: anti-mouse IgG HRP Conjugated and anti-rabbit IgG HRP conjugated (R&D Systems).

Supplementary Figures

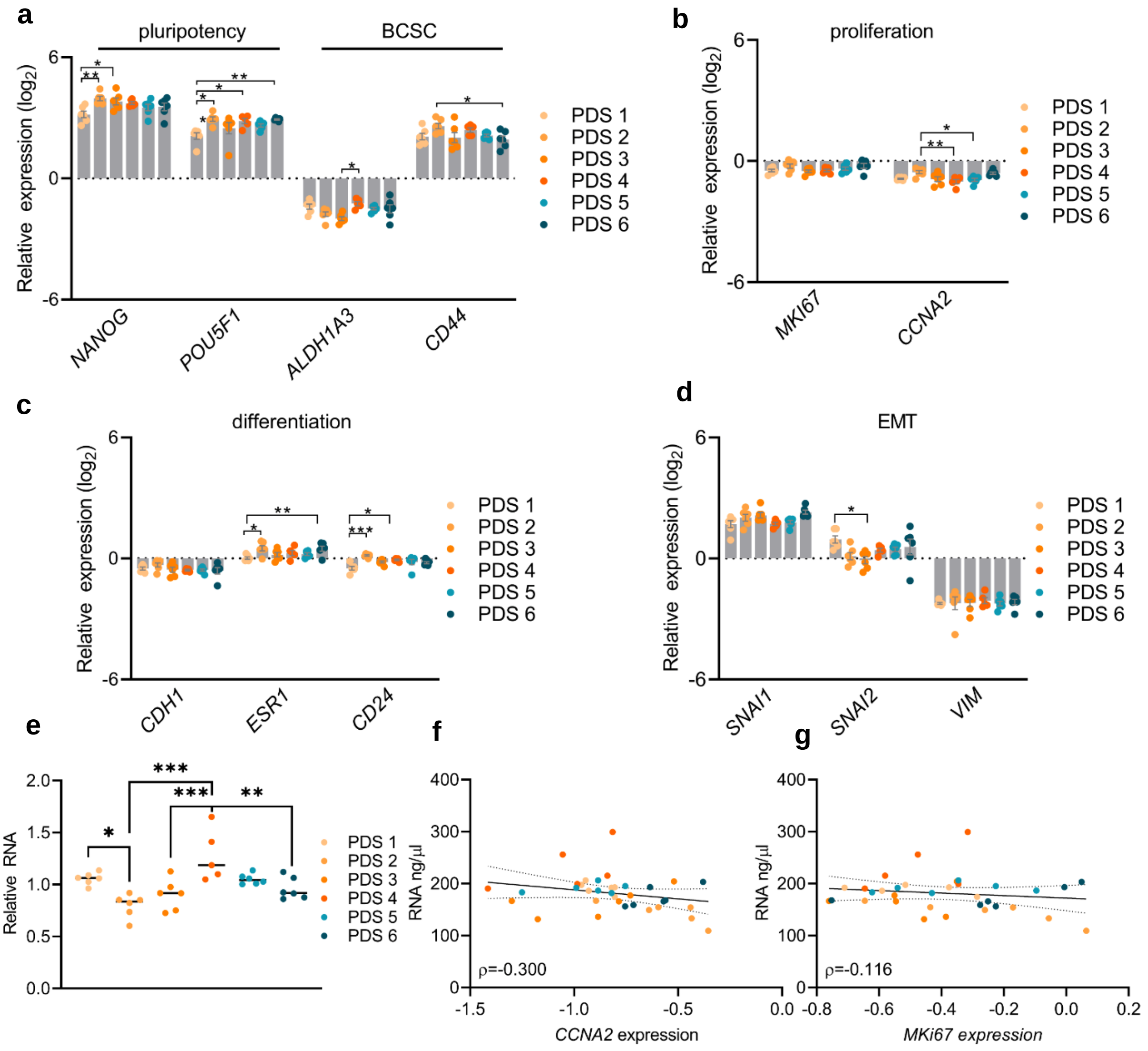

Supplementary Figure 1. Inter- versus intra- heterogeneity between patient-derived scaffold slices. (a-d) Scatterplots demonstrate gene expression analysis of biological patient-derived scaffolds (PDS) replicates. Quantitative PCR data shows the induced gene regulation of cells cultured in PDSs. Each dot represents the relative expression of cells cultured in each PDS slice compared to 2D cultures in log2-scale. One-Way ANOVA with Tukey's multiple comparison test was done between all the groups combinations (*$p \leq 0.05$, **$p \leq 0.01$, ***$p \leq 0.001$). BCSC; breast cancer stem cells, EMT; Epithelial-to-mesenchymal transition. (e) Levels of RNA extracted from 6 different PDSs with 5-6 replicates each One-Way ANOVA with Tukey's multiple comparison test was done between all the groups combinations (*$p \leq 0.05$, **$p \leq 0.01$, ***$p \leq 0.001$). (f-g) Scatterplots demonstrating Spearman correlations (ρ) between RNA levels from MCF7-PDS lysates and the expression of proliferation markers (f) *CCNA2* and (g) *MKI67* for each PDS slice. Patient-derived scaffold identity is indicated by colors.

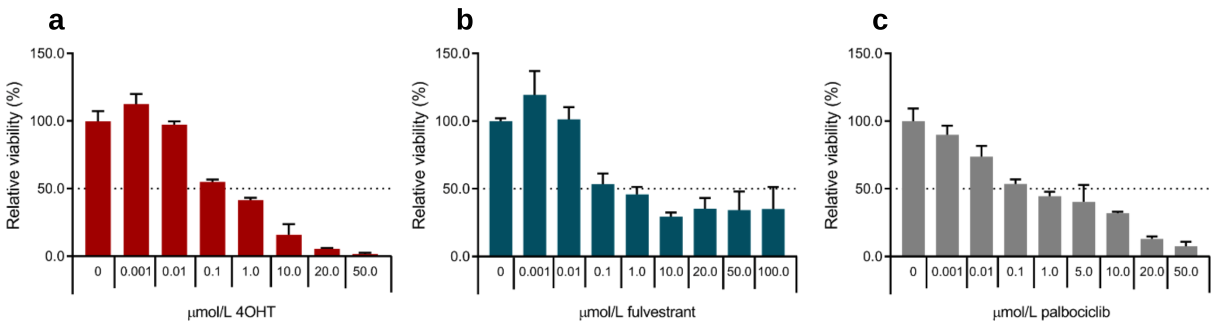

Supplementary Figure 2. Cell viability measurements after therapeutic treatment with increasing concentrations of (Z)-4-Hydroxytamoxifen, fulvestrant or palbociclib in 2D cultures. Alamar blue viability assay was performed in MCF7 cells growing in 2D cultures that were stimulated with increasing concentration of (a) (Z)-4-Hydroxytamoxifen (4OHT), (b) fulvestrant or (c) palbociclib for 96 hours. Bar-graphs represent relative cell viability compared to controls, Mean+ SEM is shown, n=3.

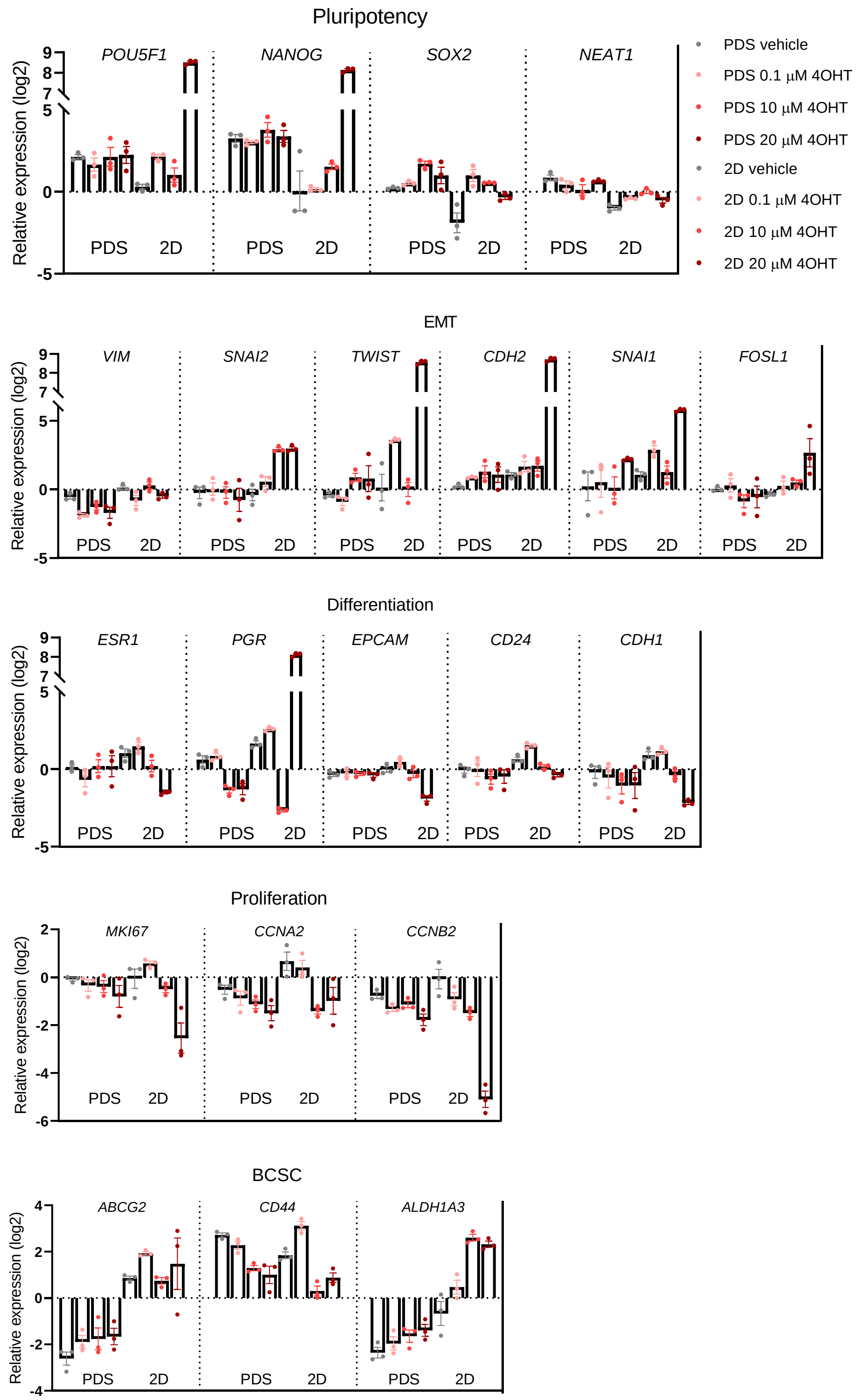

Supplementary Figure 3. Gene expression analyzes of PDS and 2D cultures with MCF7 cells after treatment with increasing concentrations of (Z)-4-Hydroxytamoxifen. Data are relative to untreated 2D cultures and expressed in log2-scale**.**

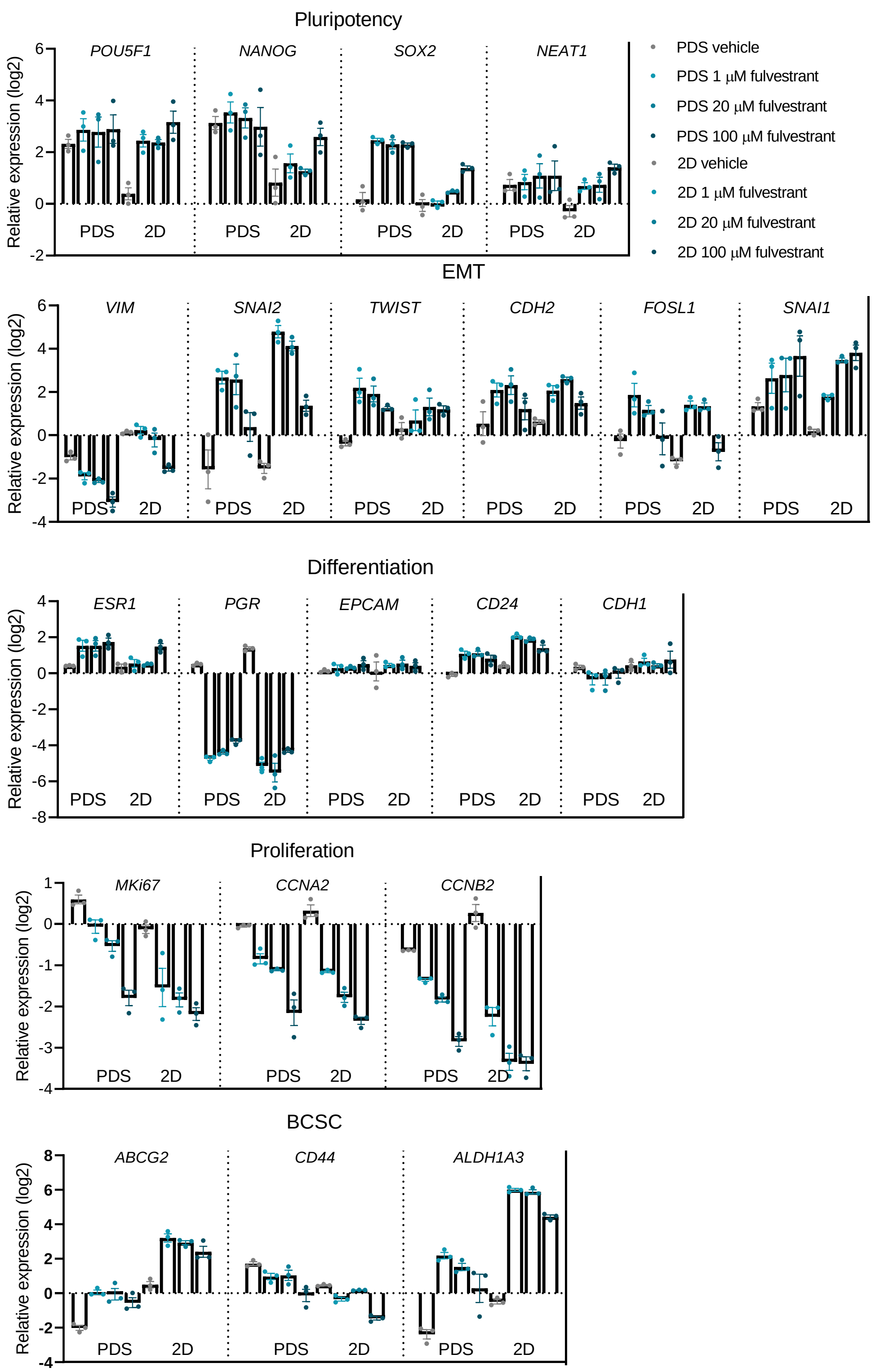

Supplementary Figure 4. Gene expression analyzes of PDS and 2D cultures with MCF7 cells after treatment with increasing concentrations of fulvestrant. Data are relative to untreated 2D cultures and expressed in log2-scale**.**

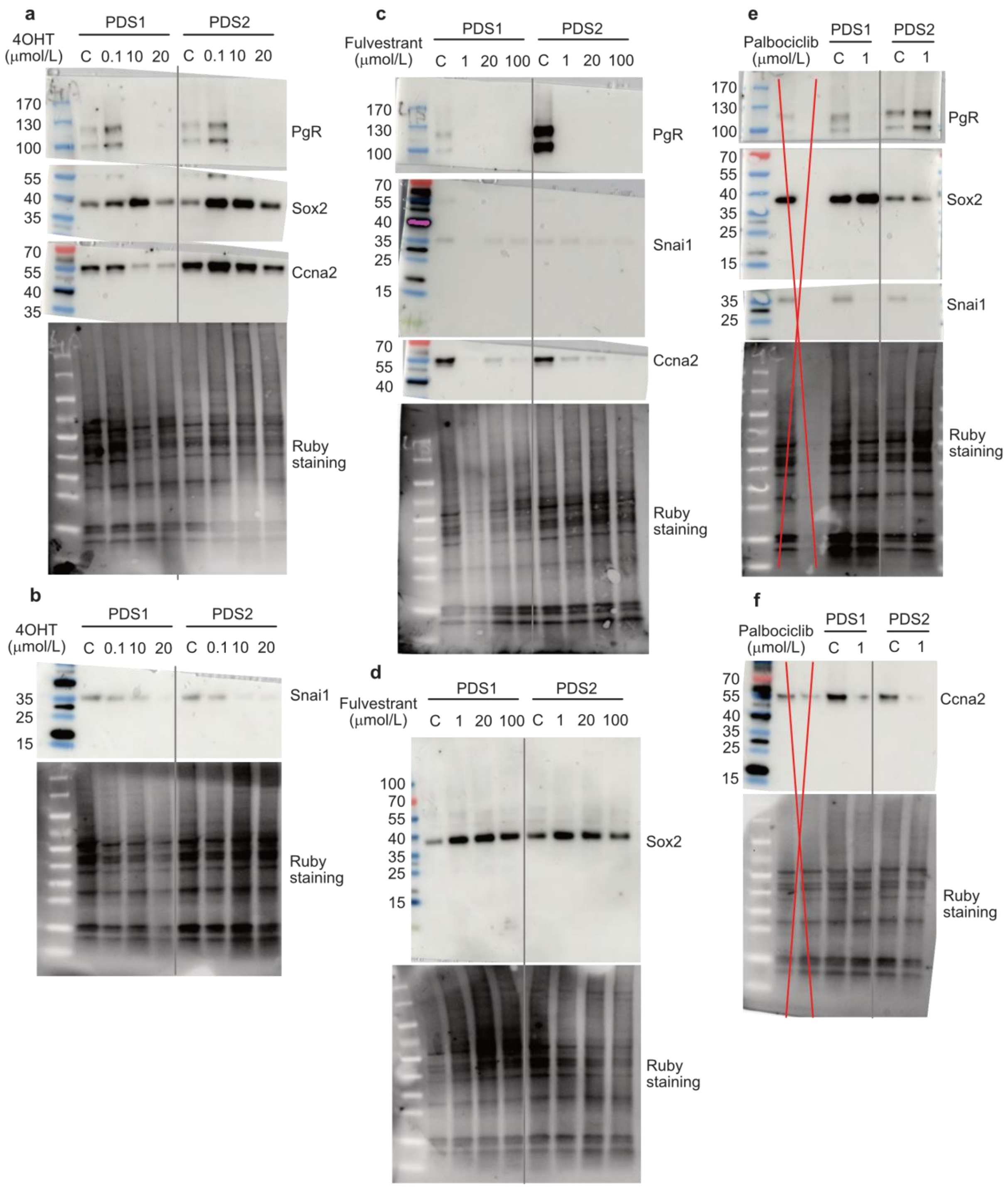

Supplementary Figure 5. Western blots showing the PgR, Sox2, Snai1 and Ccn2 levels in treated PDSs cultures. Protein extracts from MCF7-PDSs were analyzed after treatment with increasing concentrations of 4OHT (a, b), fluvestrant (c, d) and 1umol/L of palbociclib (e, f). SYPRO Ruby staining of the membrane after transference was used as a loading control.

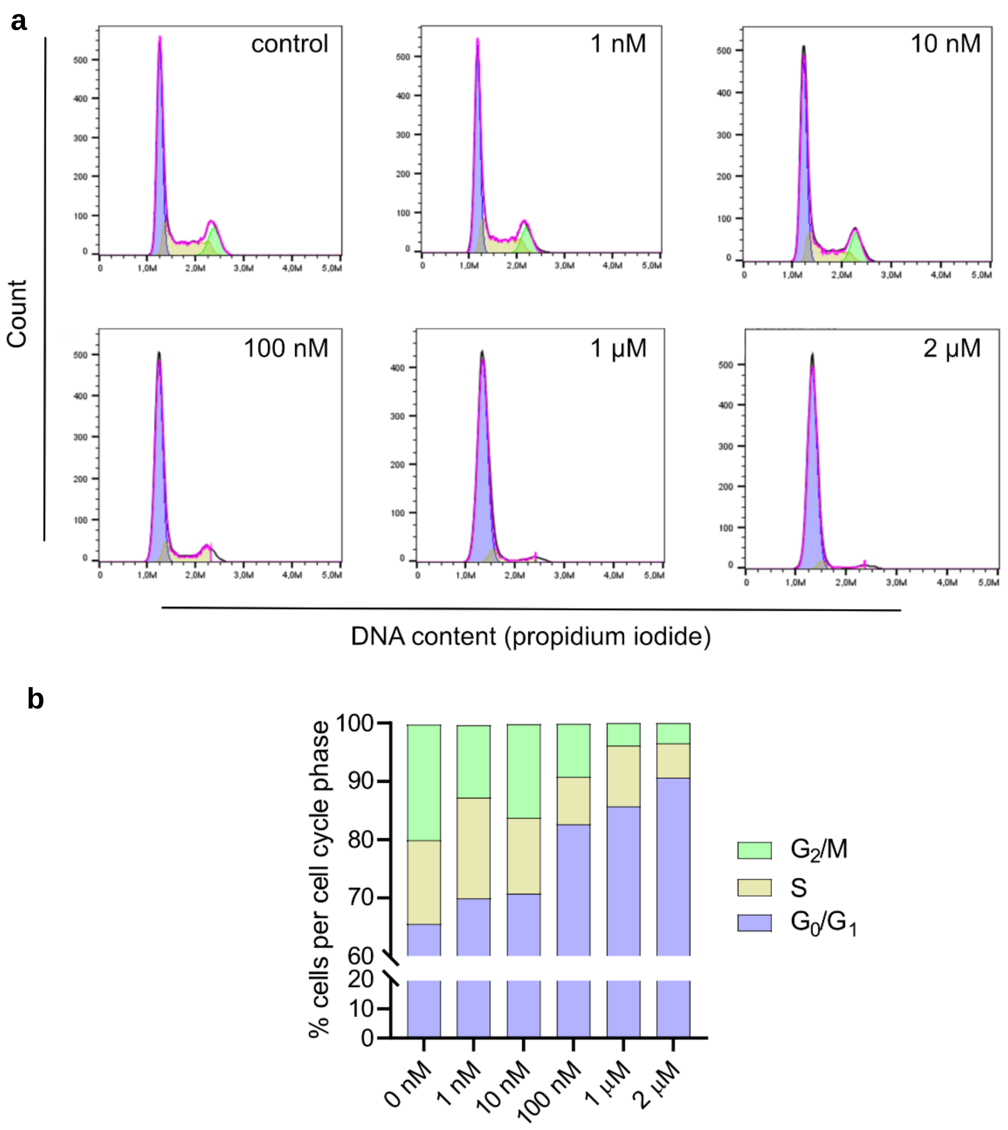

Supplementary Figure 6. Evaluation in 2D cultures of increasing concentrations of palbociclib for treatments. (a) Representative figure of cell-cycle phase distribution of propidium iodide-stained cells after treatment with palbociclib using flow cytometry. (b) Column diagram showing the percentage of cells in each phase of cell cycle after palbociclib treatment.

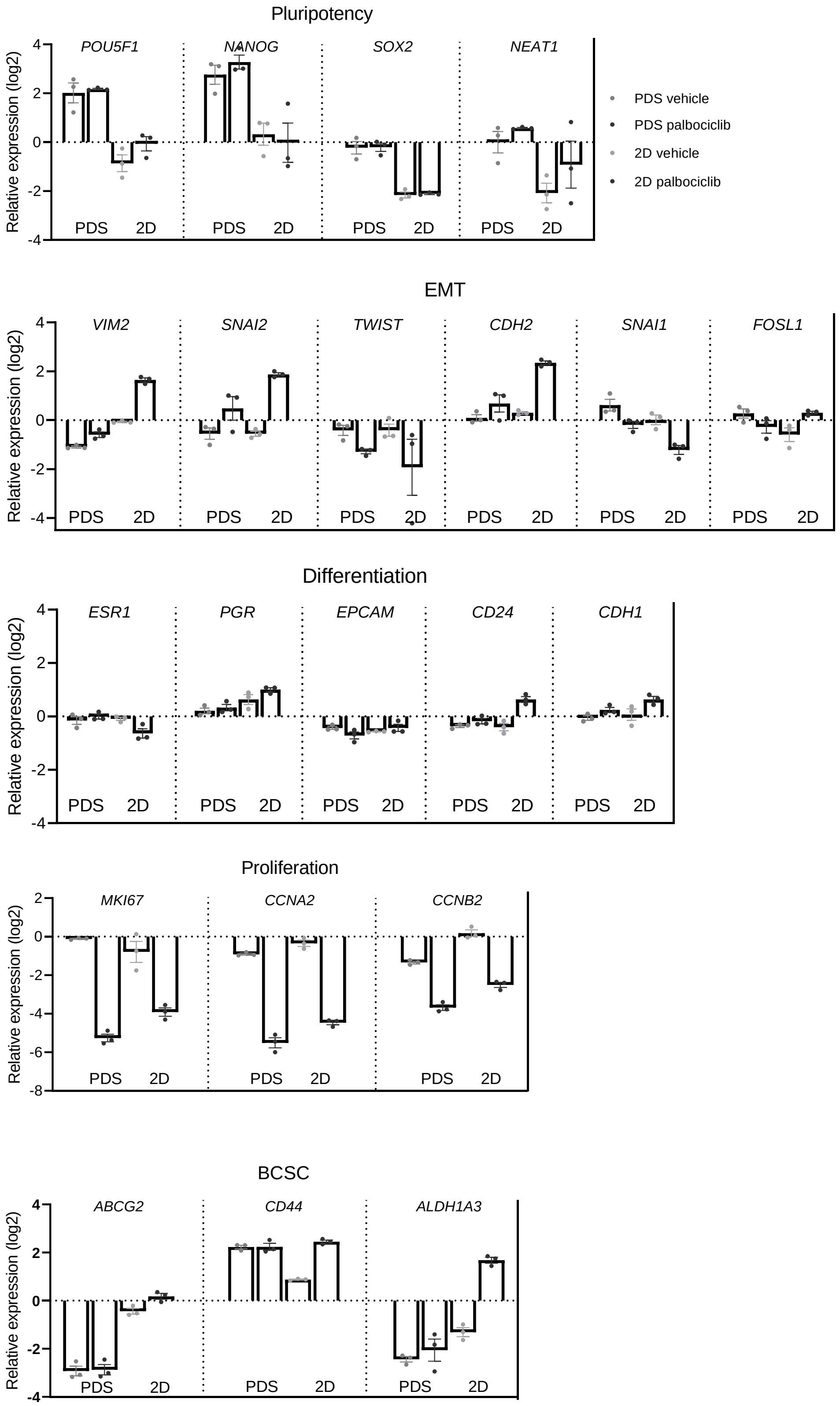

Supplementary Figure 7. Gene expression analyzes of PDS and 2D cultures with MCF7 cells after treatment with 1 µmol/L palbociclib. Data are relative to untreated 2D cultures and expressed in log2-scale**.**

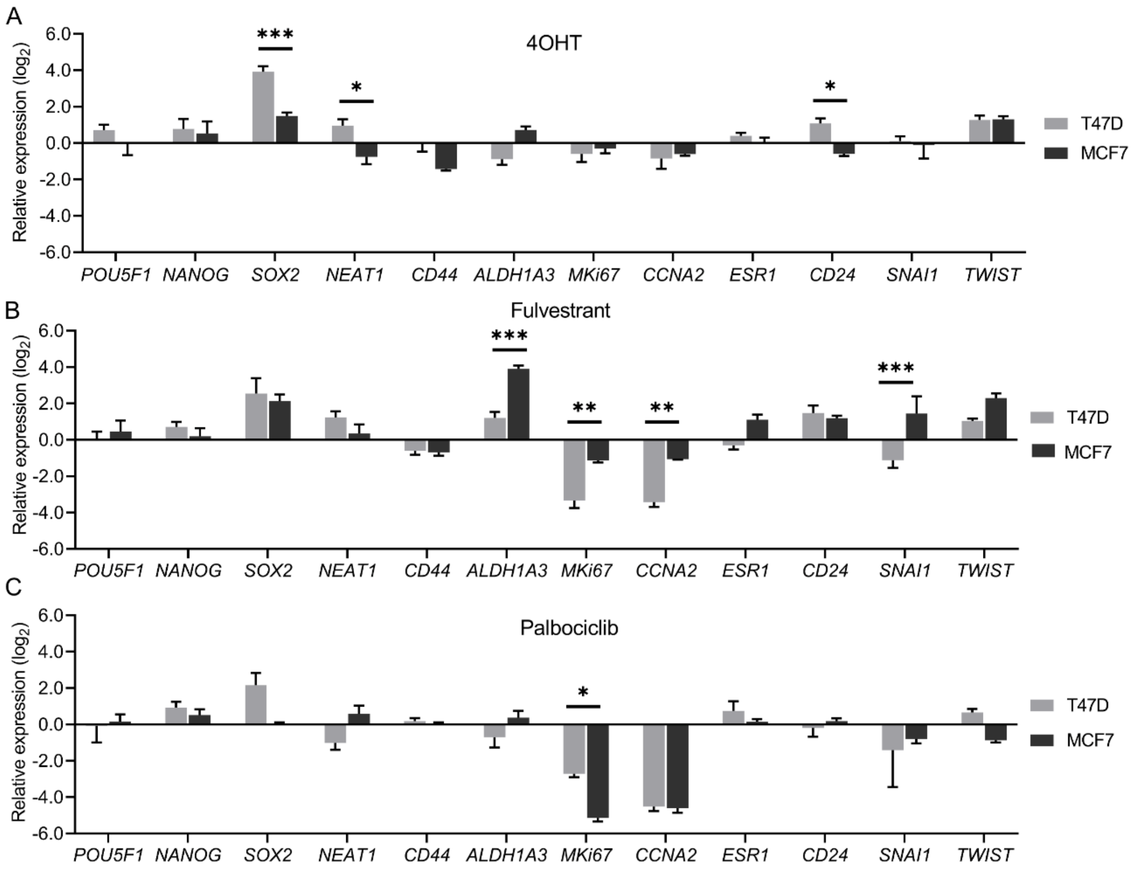

Supplementary Figure 8. T47D and MCF7 cells grown in patient-derived scaffolds induce similar gene expression changes after drug treatments. Bar-graphs showing the quantitative PCR data of PDS-induced gene expression regulation after drug treatment in T47D and MCF7 cells with (a) 10 µmol/L (Z)-4-Hydroxytamoxifen (4OHT), (b) 20 µmol/L fulvestrant or (c) 1 µmol/L palbociclib. Gene expression is relative to 2D control cultures for each cell line and expressed in log2-scale. Mean+ SEM is shown, (n=3). (*$p \leq 0.05$, **$p \leq 0.01$, ***$p \leq 0.001$, Two-Way ANOVA, with Sidak's multiple comparison test).

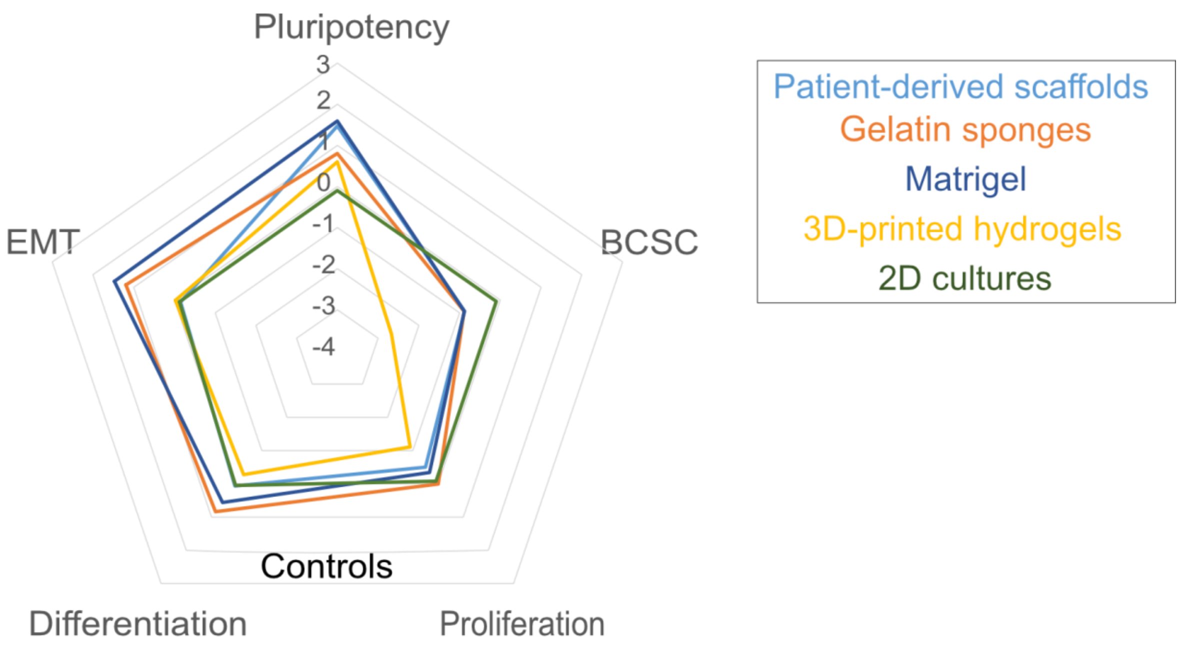

Supplementary Figure 9. Radar chart shows the main trend of the gene expression changes in pluripotency, breast cancer stem cell (BCSC), proliferation, differentiation and epithelial-to-mesenchymal transition (EMT) categories for untreated (vehicle) MCF7 cells grown in patient-derived scaffolds (PDS), gelatin sponges, matrigel and three-dimensional (3D)-printed hydrogels. Gene expression is expressed relative to 2D culture, which is included as a control and depicted in the graph. Plotted data was calculated as the average of log2-scale expression of the genes grouped in each category (categories are detailed in Supplementary Table 1) and including 3 replicates for each culture platform.

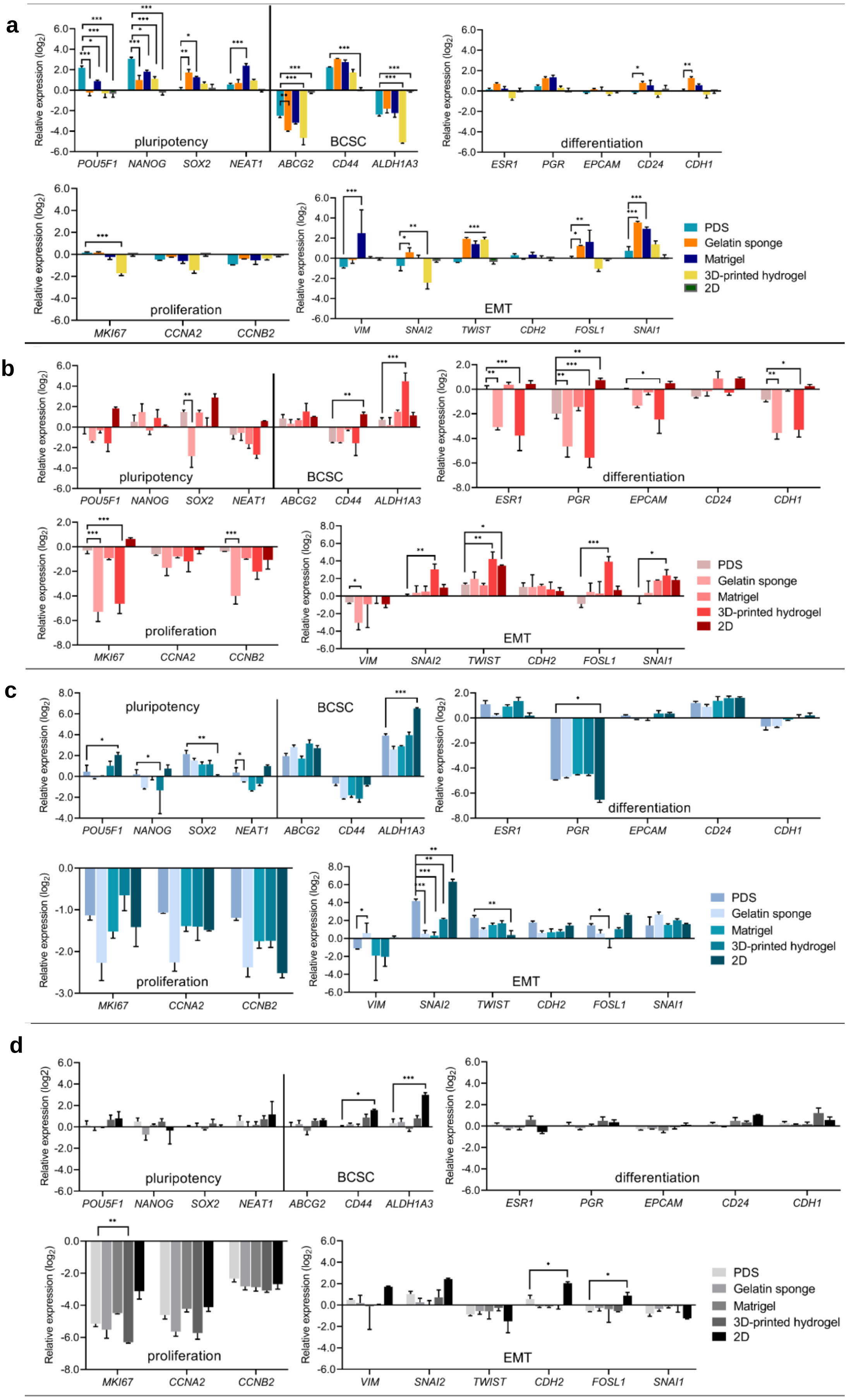
a
b
c
d
Relative expression (log2)
pluripotency
BCSC
differentiation
proliferation
EMT
POU5F1
NANOG
SOX2
NEAT1
ABCG2
CD44
ALDH1A3
ESR1
PGR
EPCAM
CD24
CDH1
MKI67
CCNA2
CCNB2
VIM
SNAI2
TWIST
CDH2
FOSL1
SNAI1
PDS
Gelatin sponge
Matrigel
3D-printed hydrogel
2D

Supplementary Figure 10. Gene expression data of cells grown in different cell culture models in controls and after treatments. Bar-graphs depicting the gene expression of MCF7 cells after three weeks of growth in gelatin sponges, matrigel, three-dimensional (3D)-printed hydrogels or 2D cultures compared to PDS, in control conditions (a) and after treatment with 10 µmol/L or 0.1 µmol/L (Z)-4-Hydroxytamoxifen (4OHT) for 3D and 2D cultures respectively (b), 20 µmol/L or 1 µmol/L fulvestrant for 3D and 2D cultures respectively (c) or 1 µmol/L palbociclib (d). BCSC, breast cancer stem cell; EMT, Epithelial-to-mesenchymal transition. Mean+ SEM is shown, n=3. (*$p \leq 0.05$, **$p \leq 0.01$ ***$p \leq 0.001$, One-way ANOVA, Dunnett's multiple comparisons test).

Supplementary Tables

Supplementary Table 1: Primer information.

Gene family	Gene	Accession number	Forward sequence (5'-3')	Reverse sequence (5'-3')
**Pluripotency**	*SOX2*	NM_003106	ACACCAATCCCATCCACACT	CCTCCCCAGGTTTTCTCTGT
	NANOG	NM_024865	CCTATGCCTGTGATTTGTGG	AAGTGGGTTGTTTGCCTTTG
	POU5F1	NM_002701	CGAAAGAGAAAGCGAACCAG	AACCACACTCGGACCACATC
	NEAT1	NR_028272.1	GCCTTCTTGTGCGTTTCTCG	CCCTCCCAGCGTTTAGC
**Proliferation**	*MKI67*	NM_004360.4	TGGGTCTGTTATTGATGAGCC	CATCAGGGTCAGAAGAGAAGC
	CCNA2	NM_001237.4	AAGACGAGACGGGTTGC	GGCTGTTTACTGTTTGCTTTCC
	CCNB2	NM_004701.3	CGACCCTTGCCACTACACTT	TGACTTCCAATACTTCATTCTCTG
**Differentiation**	*ESR1*	NM_000125.3	TGACTATGCTTCAGGCTACCAT	ACCTTTCATCATTCCCACTTC
	PGR	NM_000926.4	TAAATGAACAGCGGATGAAAGAA	CGACACAACTCCTTTTTGCCT
	EPCAM	NM_002354.2	CAGGAAGAATGTGTCTGTGAAAACT	TTCATTTCTGCCTTCATCACC
	CD24	NM_013230.2	GCTCCTACCCACGCAGATT	GGTGGTGGCATTAGTTGGAT
	CDH1	NM_004360.4	AGAGGACCAGGACTTTGACTTG	CAGAGAATCATAAGGCGGGG
**Breast cancer stem cell**	*CD44*	NM_000610.3	GAAGAAGGTGTGGGCAGAAGA	ACCATTTCCTGAGACTTGCTG
	ALDH1A3	NM_000693.2	AAAAAGAGCGAATAGCACCG	GCATAGAGGGCGTTGTAGCA
	ABCG2	NM_004827.2	GGTGGAGGCAAATCTTCGTTA	GAGTGCCCATCACAACATCA
**Epithelial-to-mesenchymal transition**	*VIM*	NM_003380.4	CAGATGCGTGAAATGGAAGA	TGGAAGAGGCAGAGAAATCC
	SNAI1	NM_005985.3	TAATCCAGAGTTTACCTTCCAGCA	AGCCTTTCCCACTGTCCTCA
	SNAI2	NM_003068.4	GCCAAACTACAGCGAACTGG	AGGAGGTGTCAGATGGAGGA
	TWIST	NM_000474.3	GGACAGTGATTCCCAGACGG	CATAGTGATGCCTTTCCTTTCAG
	CDH2	NM_001792.3	CATTATCAACCCCATCTCGG	ACTGTCCCATTCCAAACCTG
	FOSL1	NM_001300855.2	GCAGGCGGAGACTGACAA	GGGGAAAGGGAGATACAAGG
**Reference genes**	*GAPDH*	NM_001289745.1	CCCACTCCTCCACCTTTGAC	GCCAAATTCGTTGTCATACCAGG
	EIF1	NM_005801.3	TCGTATGTCCGCTATCCAGA	TAAGGGTCTTCCTGCCGTTT
	YWHAZ	NM_001135699.1	ACGCCTCACTCCCGTTT	CTGGATGTTCTGCTGGCTC
	RPS26	NM_001029.5	GATGCGTGCCCAAGGAC	CAGGTCTAAATCGGGGTGG
	RPL7	NM_001014.5	CCTGAGAAAGAAGTTTGCCA	CTTGCCATCCTCGCCATT
	RPS10	NM_001014.4	AGCCGCAGAGATGTTGATG	CCTCGGGACTTGAGAGACTG

Supplementary Table 2. Histo-pathological characteristics of the included breast cancer samples used to generate cell-free patient-derived scaffolds. IDC= Invasive ductal carcinoma; ILC= Invasive lobular carcinoma.

**Number of patients**	21
**Histological type**	
IDC	16
ILC	2
Other	3
**Histological grade**	
Grade I	2
Grade II	8
Grade III	9
Missing	2
**KI67 staining**	
≤15%	5
15-60%	10
≥60%	4
Other	2
**Estrogen receptor**	
Positive	14
Negative	5
Missing	2
**Progesterone receptor**	
Positive	15
Negative	4
Missing	2
**HER2-status**	
Positive	2
Negative	17
Missing	2
